# Spontaneous Acquired Diaphragmatic Hernia in an Elderly Female: A Case Report of Abdominal Mesh Repair and Diagnostic Challenges

**DOI:** 10.7759/cureus.83376

**Published:** 2025-05-02

**Authors:** Priyanka Prakash, Kuberan Krishnan, Magesh Chandran, Madan Sundar

**Affiliations:** 1 General Surgery, Sree Balaji Medical College & Hospital, Chennai, IND; 2 Surgery, Sree Balaji Medical College & Hospital, Chennai, IND

**Keywords:** diaphragmatic hernias, hiatus hernia, mesh repair for diaphragmatic hernia transabdominal approach, spontaneous acquired diaphragmatic hernia, transabdominal approach

## Abstract

Spontaneous acquired diaphragmatic hernia is an exceptionally rare condition. Clinical presentation is always subtle and non-specific, thus contributing to the delay in early diagnosis and treatment, which, on the other hand, is vital to prevent complications such as respiratory distress and organ strangulation from its occurrence. Thoracic and abdominal approaches are practiced, wherein the transabdominal approach gets extra points for the ease of performing this surgery when it comes to field visualization and managing intra-abdominal adhesions. This case report elaborates on one successful story of an old lady who underwent mesh repair via transabdominal approach, with an uneventful postoperative recovery. We have discussed the challenges in diagnosing the condition, the role of imaging in confirming the diagnosis, and the advantages of the transabdominal approach. By reviewing this case and relevant literature, we aim to create awareness about this rare entity and highlight the value of early diagnosis and appropriate treatment measures at the right time, potentially improving patient outcomes.

## Introduction

Diaphragmatic hernia, a rare surgical emergency, typically arises from trauma, most often blunt force injuries sustained in motor vehicle accidents. When this occurs, abdominal organs move into the chest cavity [1]. Non-hiatal diaphragmatic hernias are rare in adults. Congenital forms, usually seen in children, result from anatomical defects in the diaphragm, allowing passage between the chest and abdomen. In contrast, acquired non-hiatal hernias, accounting for only 1%-7% of cases, are caused by traumatic injury, often blunt force, which damages the diaphragm [2]. Spontaneous acquired diaphragmatic hernia, especially when there is no history of trauma, is an exceptionally rare condition, accounting for <1% of all diaphragmatic hernias, with only a few dozen cases documented in medical literature [3,4]. Spontaneous acquired diaphragmatic hernia occurs when abdominal organs are pushed into the chest cavity through a diaphragmatic tear, typically caused by a sudden rise in intra-abdominal pressure. This pressure surge can be triggered by activities like strenuous exercise, childbirth, coughing, vomiting, or straining during bowel movement [5].

## Case presentation

**Figure 1:**
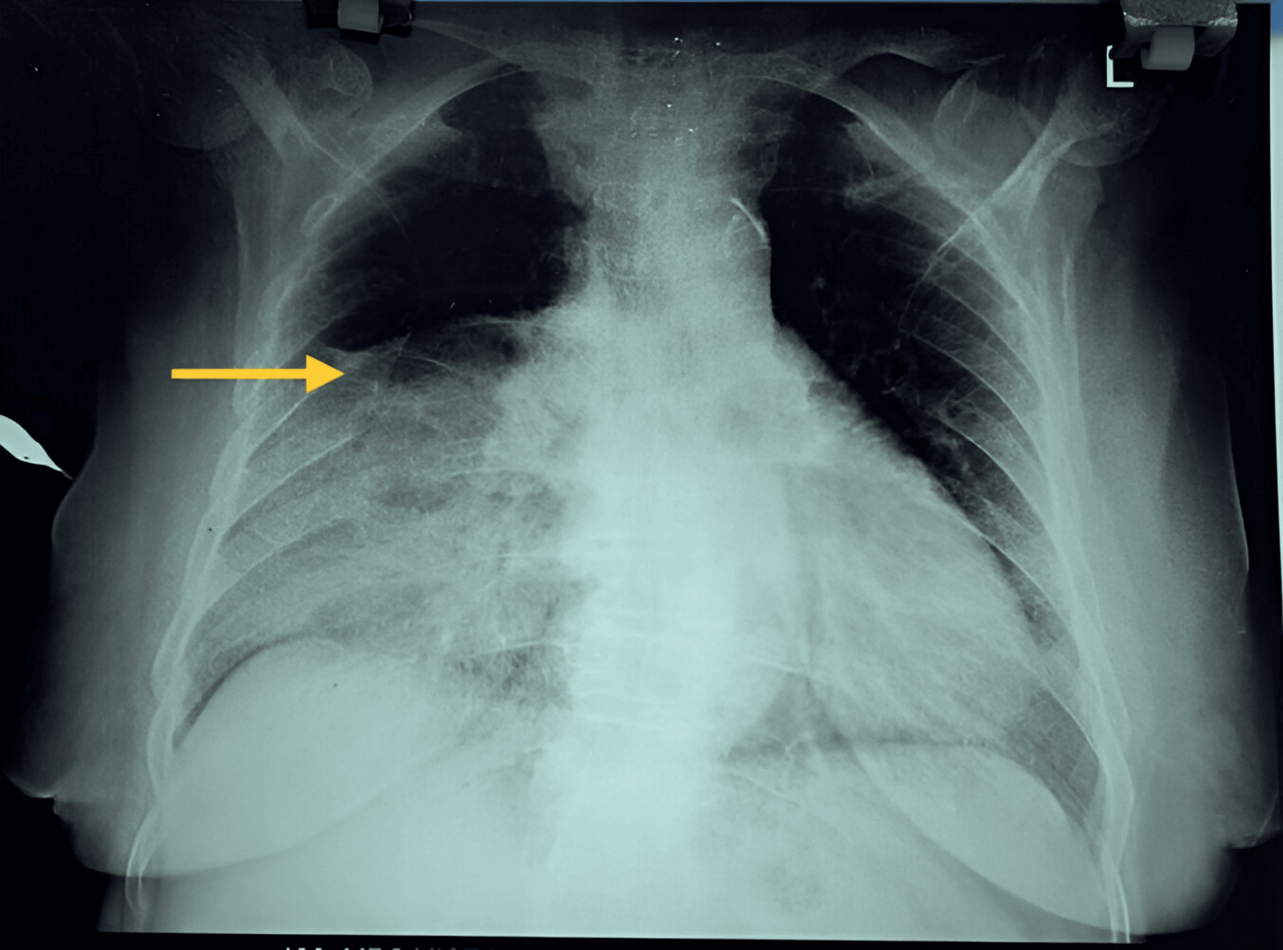
Chest X-ray showing non-homogeneous opacity in the right inframammary region, lower limit of visualized right lung parenchyma (yellow arrow), and mild mediastinal shift to the left side.

A 60-year-old lady presented with intermittent epigastric pain and postprandial discomfort for a period of six months. She had no history of recent trauma, no known comorbid condition, and was not on any medication. There was no previous surgical history. The BMI of the patient was 31.2. On examination, air entry on the right inframammary and infra-axillary regions was decreased when compared to the other sides, while bowel sounds were heard in the thoracic cavity, with mild epigastric region and right hypochondriac region tenderness. At this point, a diaphragmatic hernia was suspected. A routine blood workup was performed and was within normal limits. Chest X-ray revealed non-homogeneous opacity in the right inframammary region, with mild mediastinal shift to the left side (Figure 1).

**Figure 2:**
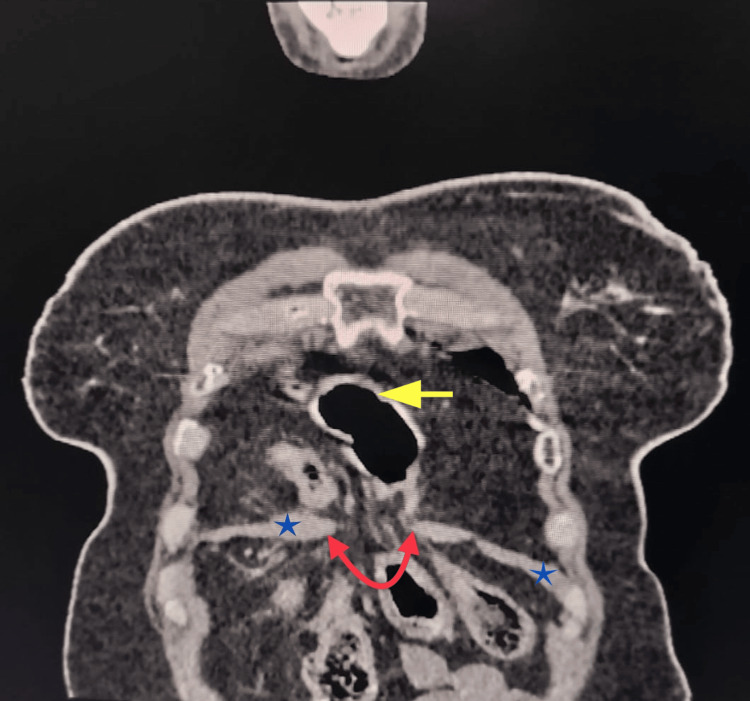
Coronal view of CT showing diaphragmatic defect (red arrow) through which the abdominal content (large bowel, marked with a yellow arrow) has herniated into the thoracic cavity, while the diaphragm is marked with blue asterisks.

**Figure 3:**
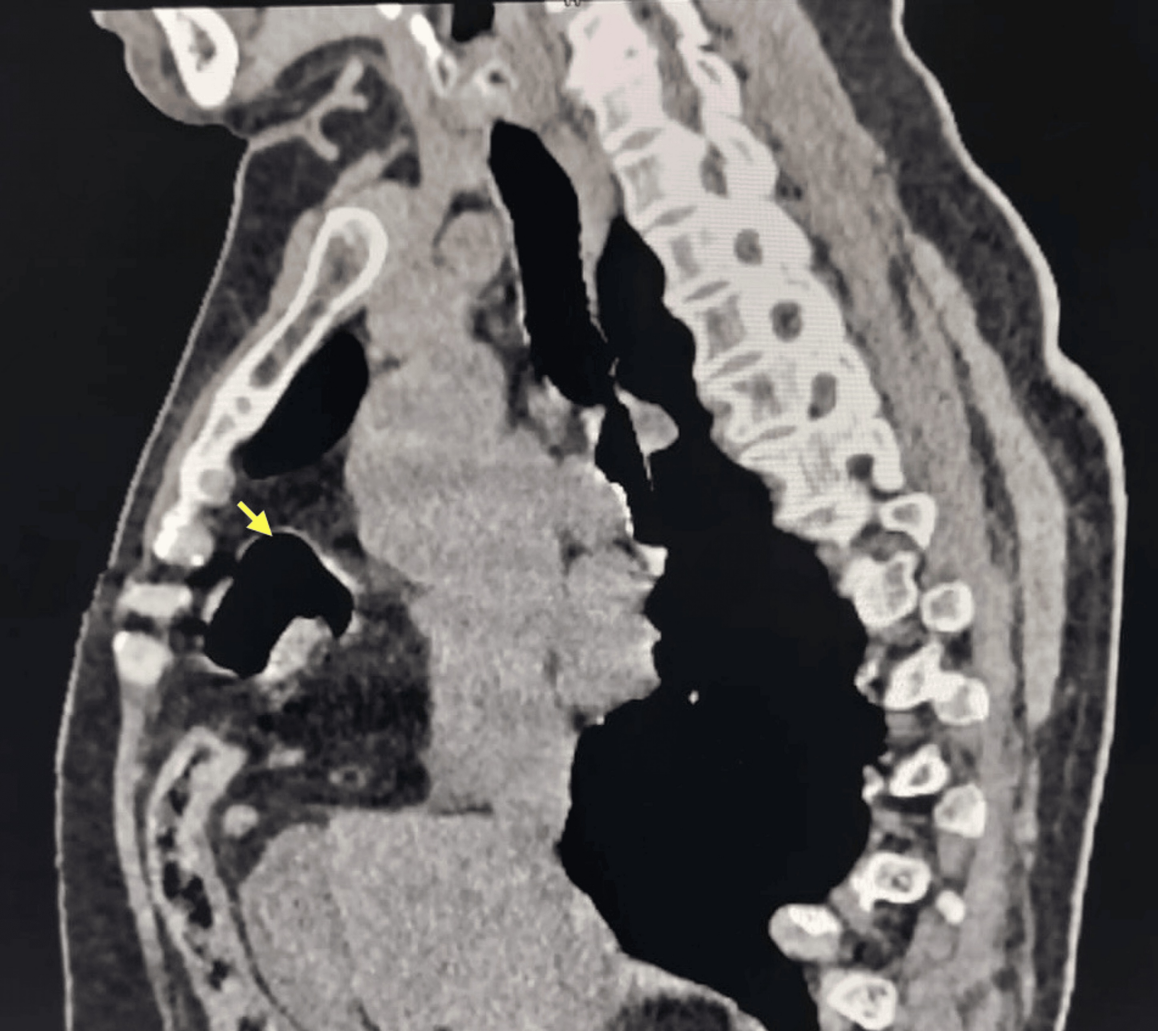
Sagittal view of CT showing the herniation of abdominal content (large bowel, marked with a yellow arrow) into the thoracic cavity.

CT confirmed a large diaphragmatic defect of size approximately 3.5 cm at the midline of the anterior diaphragmatic crura (Figures 2, 3). A part of the transverse colon and omentum was identified as the herniated content. No signs of strangulation or ischemia of the herniated bowel were observed.

Pulmonary function test revealed restrictive lung disease due to compression by herniated abdominal contents.

Given the acquired spontaneous nature of the hernia, chronic nature of the complaint and the size of the defect (approximately 3.5 cm in diameter) with transverse colon as the hernial content, dense adhesions were expected and hence open abdominal approach was chosen for better access and control during organ reduction and defect repair. Mesh reinforcement was planned, as the size of the defect was large, which precluded primary closure without tension. Since the content was transverse colon, the defect size was expected to be larger than measured; hence open abdominal approach would provide excellent exposure.

**Figure 4:**
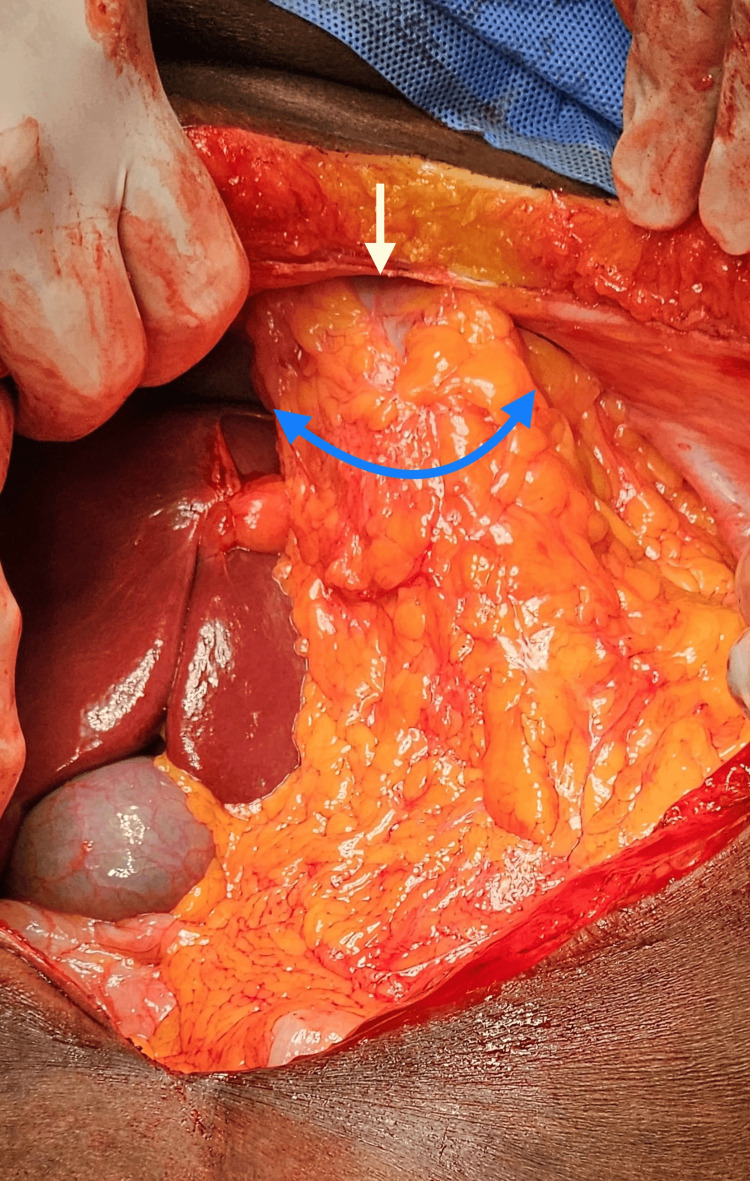
Intraoperative image showing the diaphragmatic defect (white arrow) through which the bowel and omentum (blue arrow) have herniated into the thoracic cavity.

The patient was in supine position, and general anesthesia was given with single lung ventilation (left lung, since the hernia was on the right side, with the herniated content compressing the right lung). A midline laparotomy incision was made to access the abdominal cavity. Herniated organs (omentum and transverse colon) were carefully reduced into their anatomical positions (Figure 4).

**Figure 5:**
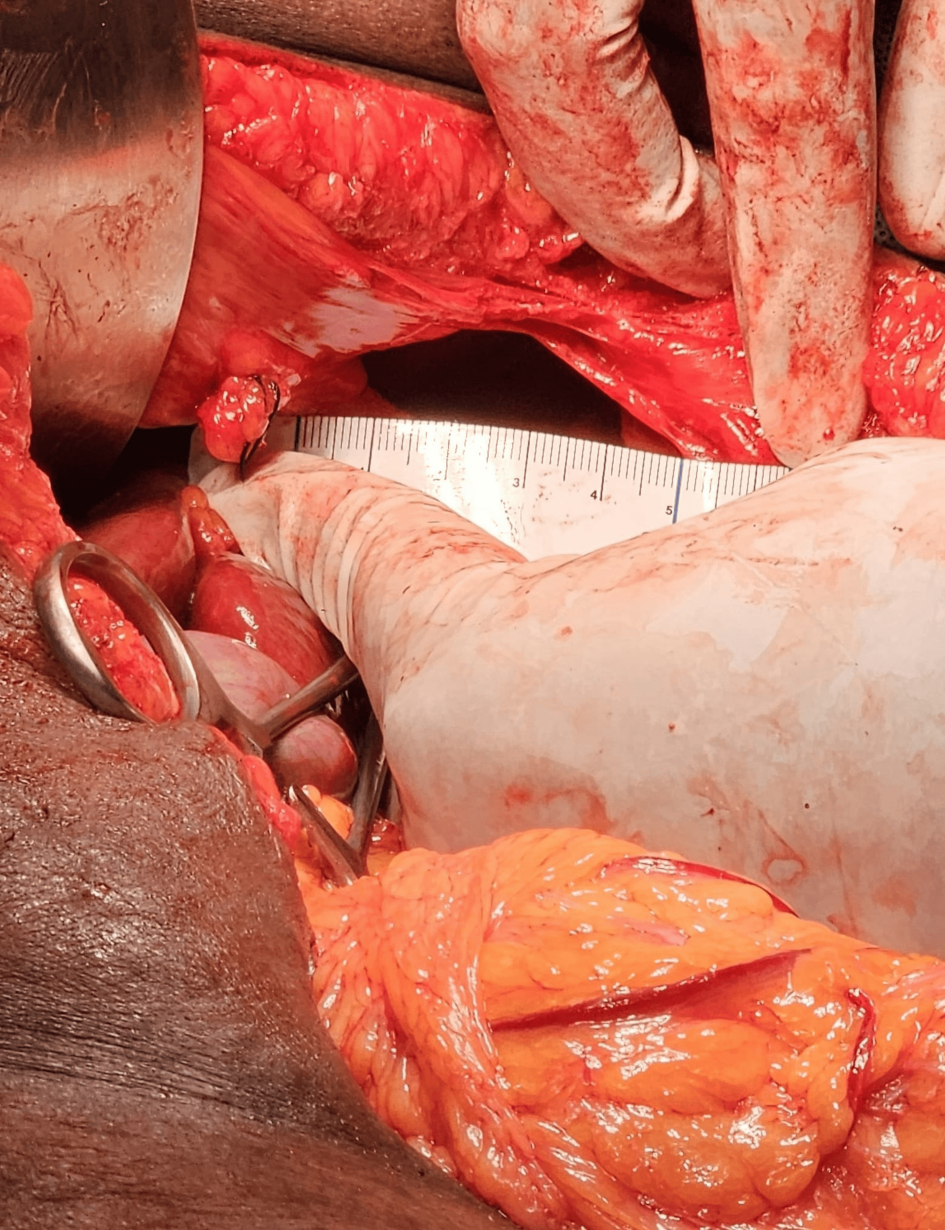
Intraoperative image showing transverse diameter of the diaphragmatic defect.

**Figure 6:**
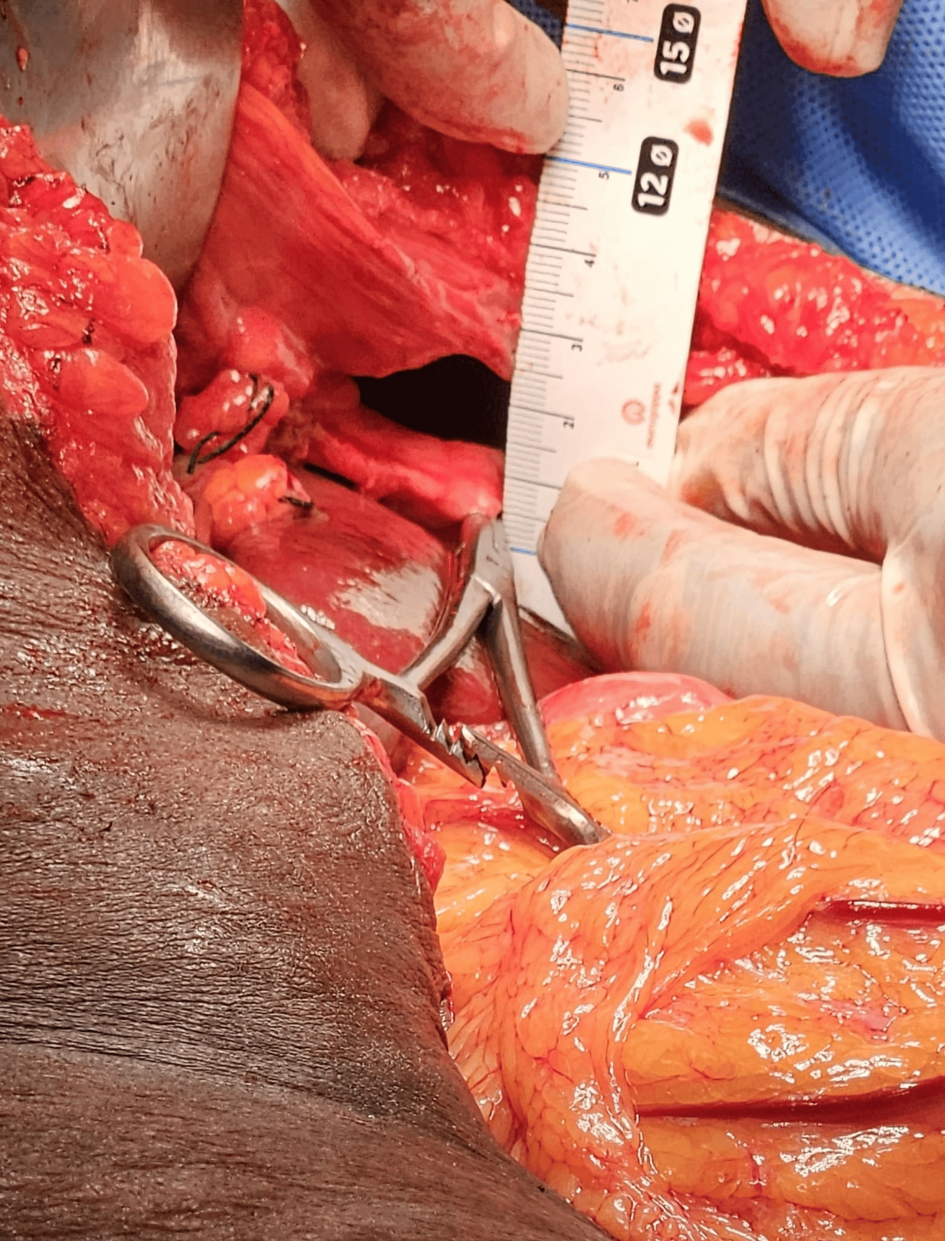
Intraoperative image showing vertical diameter of the diaphragmatic defect.

Adhesions between herniated organs and surrounding tissues were meticulously dissected to prevent injury to visceral structures. The diaphragmatic defect was identified as 5 cm (transverse; Figure 5) x 1.5 cm (vertical; Figure 6) in diameter with thin edges.

**Figure 7:**
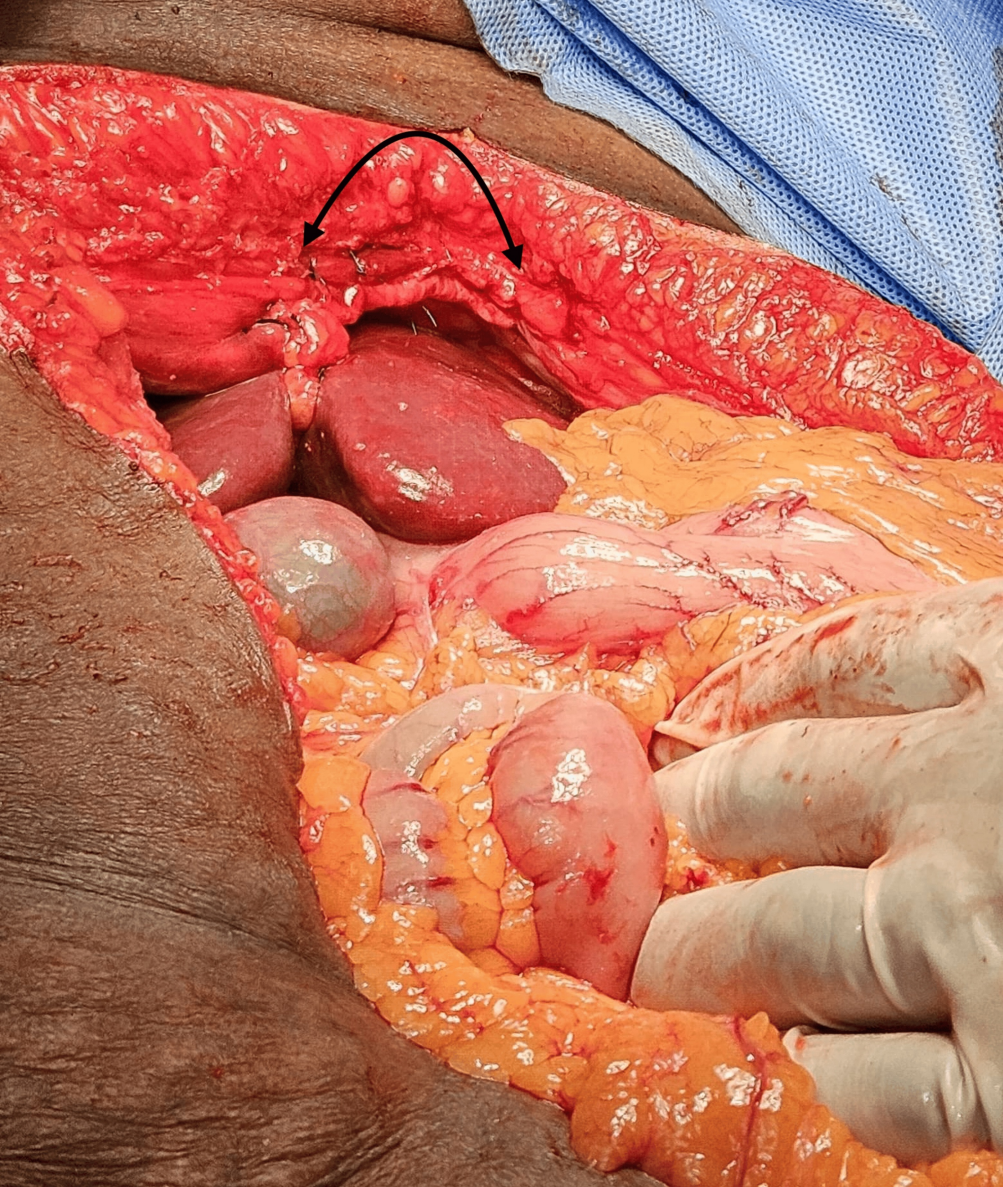
Intraoperative image showing primary closure (black arrow) of the defect with non-absorbable suture.

Non-absorbable sutures using 2-0 Prolene (round body needle) for initial approximation of defect edges to minimize tension were performed (Figure 7).

**Figure 8:**
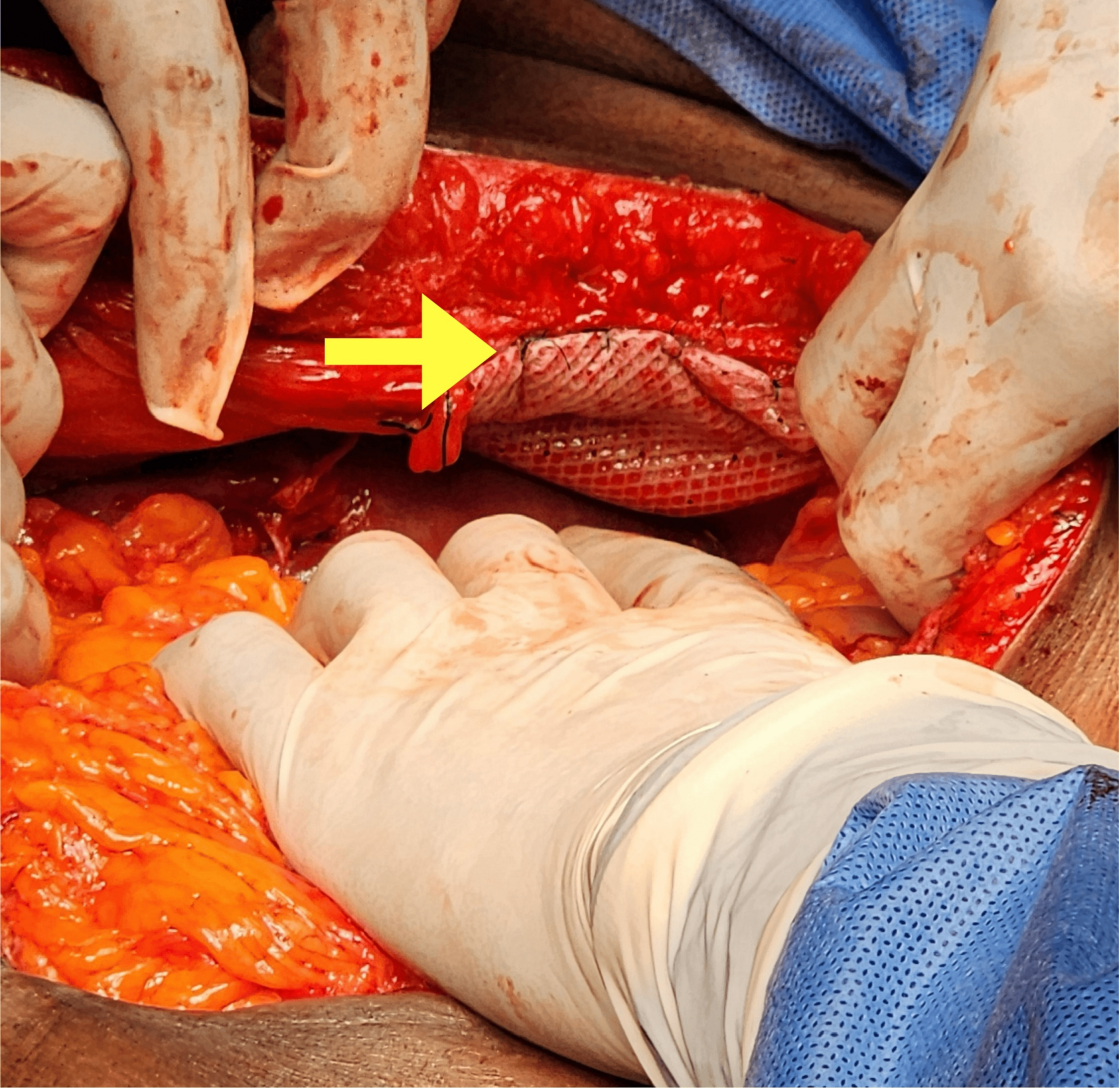
Intraoperative image showing the composite mesh (yellow arrow) that was fixed over the closed diaphragmatic defect.

A synthetic (12 x 12 cm) composite mesh (Figure 8) was tailored to cover the defect. Along the anterior border of the diaphragmatic crura, mesh was fixed using interrupted horizontal mattress sutures (2-0 Prolene), while simple interrupted sutures were used around the defect margin to ensure stability. Care was taken to avoid excessive tension on the diaphragm and mesh.

Following mesh fixation, since pneumothorax/pleural effusion is an expected complication due to the void created after reducing the hernia, the intercostal drain was inserted prophylactically. The abdominal cavity was irrigated with saline. The wound was closed in layers after ensuring hemostasis.

Following surgery, the patient was extubated in the operation theater and then shifted to the ICU for close monitoring. Pain management included epidural analgesia for optimal respiratory function. Chest physiotherapy and incentive spirometry were initiated on postoperative day one to prevent pulmonary complications. There was no collection; hence, the intercostal drain was removed on postoperative day two. No major intraoperative or immediate postoperative complications occurred. The patient was discharged on postoperative day seven after demonstrating improved respiratory function and tolerance to oral intake. About three months after surgery, the patient reported significant symptomatic improvement and quality of life.

## Discussion

While a patient's history and physical examination can raise suspicion for a diaphragmatic hernia, imaging is essential for confirmation. Prompt diagnosis is crucial, as delays increase the risk of serious complications such as strangulation, perforation, or compression of the lungs, causing respiratory distress. Clinicians should be alert for historical indicators such as a pre-existing diaphragmatic defect, sudden shortness of breath following increased abdominal pressure, or vomiting accompanied by dyspnea. Of the many non-specific, common clinical presentations, most commonly patients present with pain in the chest or abdomen, breathing difficulties, vomiting, and nausea [6]. Key physical examination findings suggestive of a diaphragmatic hernia include reduced or absent breath sounds and the presence of bowel sounds within the thoracic cavity [6]. Imaging studies available at the causality include chest X-ray, ultrasonography, CT, MRI, and contrast studies. Of these, chest radiography, point-of-care ultrasonography, and CT are the most readily accessible [7]. Due to the limitations of chest X-rays, which only detect 25%-50% of diaphragmatic hernias, roughly 66% of these cases are missed during the patient's initial evaluation [8]. Chest X-ray findings include blunting of the costophrenic angle, elevation of the hemidiaphragm, mediastinal shift, distortion of the diaphragmatic border, pleural effusion, presence of bowel in the thorax, or migration of the Ryles tube into the thoracic cavity. Ultrasonography is limited by artifacts secondary to pulmonary and intestinal air and acoustic shadowing from the ribs [9]. CT is the preferred investigation of the many imaging techniques available in the ED for diagnosing diaphragmatic hernia due to its precision. However, surgical exploration remains the definitive method for diagnosis [10]. Surgical intervention is the definitive treatment modality for diaphragmatic herniation. Preoperatively, supplemental oxygen and nasogastric decompression may be employed for symptom palliation. Open abdominal approach provides excellent exposure for organ reduction and mesh placement [11]. In this study [11], of the 23 cases included, herniated contents were successfully reduced in 20 cases, while three cases had a difficult and prolonged intraoperative period due to severe adhesion of the herniated contents to the hernial sac. During such attempts, damage to the gastric wall, seromuscular rupture of the small bowel, and rupture of the splenic capsule occurred intraoperatively. Keeping in mind the possibility of bowel wall injury during organ reduction and the content being the transverse colon for this patient, in order to avoid such risks as severe adhesions, an open abdominal approach was chosen over laparoscopy. In this case, pneumothorax and pleural effusion were excluded preoperatively, thereby obviating unnecessary needle thoracostomy or subsequent tube thoracostomy, and minimizing patient morbidity [12]. However, given the risk of these complications due to the void created after reduction of the hernia, an implantable cardioverter defibrillator was inserted prophylactically following mesh fixation. It was removed on postoperative day two after confirming the absence of any collection.

This case contributes to the limited body of literature on spontaneous acquired diaphragmatic hernia, a rare condition accounting for less than 1% of all diaphragmatic hernias, with only a few cases reported in non-traumatized, otherwise healthy adults [3,4]. Most documented cases involve left-sided defects, making this right-sided presentation even more unusual [2,3]. The defect location and involvement of the transverse colon further underscore the anatomical and surgical complexity. The size of the diaphragmatic defect in this case, measuring approximately 5 cm transversely, was notably larger than what is typically reported in spontaneous hernias, which often range between 2 and 4 cm in diameter [4,11]. In Liu et al.’s series of 23 patients with chronic traumatic diaphragmatic hernias, many defects were under 4 cm and managed laparoscopically [11]; however, larger defects with dense adhesions required conversion to open surgery. This report illustrates the rationale for choosing an open abdominal approach in chronic, large-volume hernias with suspected adhesions and colonic involvement, which may preclude tension-free primary closure. Additionally, the use of prophylactic intercostal drainage after mesh placement helped mitigate pulmonary complications such as pneumothorax and effusion, which are recognized risks after large-volume hernia reduction [12]. By detailing diagnostic challenges, defect size considerations, and intraoperative decision-making, this case enhances current understanding and offers practical guidance in the management of rare spontaneous diaphragmatic hernias.

## Conclusions

This case highlights the successful management of a spontaneous acquired diaphragmatic hernia in a 60-year-old female using an open abdominal approach with mesh repair. The procedure provided a durable closure of a large defect while minimizing postoperative complications. Early diagnosis and tailored surgical planning are essential for optimal patient outcomes in such complex cases. Expected pulmonary complications such as atelectasis were prevented from occurring by initiating chest physiotherapy during the early postoperative period.

## References

[1] Kearney PA, Rouhana SW, Burney RE (1989). Blunt rupture of the diaphragm: mechanism, diagnosis, and treatment. Ann Emerg Med.

[2] Portelli M, Bugeja M, Cini C (2021). Left-sided Bochdalek's hernia in a young adult: a case report and literature review. Surg J (N Y).

[3] Zaidi ZA, Tebha SS, Sethar SS, Mishra S (2021). Incidental finding of right-sided idiopathic spontaneous acquired diaphragmatic hernia. Cureus.

[4] Losanoff JE, Edelman DA, Salwen WA, Basson MD (2010). Spontaneous rupture of the diaphragm: case report and comprehensive review of the world literature. J Thorac Cardiovasc Surg.

[5] Hamoudi D, Bouderka MA, Benissa N, Harti A (2004). Diaphragmatic rupture during labor. Int J Obstet Anesth.

[6] Bekassy SM, Dave KS, Wooler GH, Ionescu MI (1973). "Spontaneous" and traumatic rupture of the diaphragm: long-term results. Ann Surg.

[7] Eren S, Kantarci M, Okur A (2006). Imaging of diaphragmatic rupture after trauma. Clin Radiol.

[8] Sandstrom CK, Stern EJ (2011). Diaphragmatic hernias: a spectrum of radiographic appearances. Curr Probl Diagn Radiol.

[9] Gelman R, Mirvis SE, Gens D (1991). Diaphragmatic rupture due to blunt trauma: sensitivity of plain chest radiographs. AJR Am J Roentgenol.

[10] McDonald AA, Robinson BR, Alarcon L (2018). Evaluation and management of traumatic diaphragmatic injuries: a practice management guideline from the Eastern Association for the Surgery of Trauma. J Trauma Acute Care Surg.

[11] Liu Q, Luan L, Zhang G, Li B (2021). Treatment of chronic traumatic diaphragmatic hernia based on laparoscopic repair: experiences from 23 cases. Front Surg.

[12] McIndoe GA, Hopkins NF (1986). 'Spontaneous' rupture of the diaphragm. Postgrad Med J.

